# Blastoid Mantle Cell Lymphoma Presenting as an Oropharyngeal Mass

**DOI:** 10.7759/cureus.56378

**Published:** 2024-03-18

**Authors:** Sharina Macapagal, Chalothorn Wannaphut, Toshiaki Takahashi, Thanaboon Yinadsawaphan, Yoshito Nishimura, Jared Acoba

**Affiliations:** 1 Internal Medicine, University of Hawaii John A. Burns School of Medicine, Honolulu, USA; 2 Hematology and Oncology, The Queen's Medical Center, Honolulu, USA

**Keywords:** oropharyngeal mass, head and neck lymphoma, non-hodgkin lymphoma, blastoid variant, mantle cell lymphoma

## Abstract

Blastoid mantle cell lymphoma (MCL) is an extremely rare neoplasm with a dismal prognosis. MCL with an initial presentation in the oral cavity has been rarely reported. This report describes a 75-year-old male who presented with an oropharyngeal mass causing dysphonia and intermittent hypoxia. A biopsy and immunophenotyping confirmed MCL, favoring the blastoid variant. Imaging showed a 4.2 cm left oropharyngeal polypoid mass with extensive lymphadenopathy. His prognosis was considered unfavorable with elevated Ki-67 index, blastoid morphology, and p53 positivity of malignant cells. There was no central nervous system involvement. He received palliative radiation, resulting in profound tumor reduction and resolution of symptoms. An intensive chemoimmunotherapy was not deemed beneficial due to age, comorbidities, absence of TP53 mutation, and a personal preference for a less aggressive treatment. This case highlights the importance of risk-adapted and personalized management approaches in a very unique presentation of blastoid MCL.

## Introduction

Mantle cell lymphoma (MCL) is a heterogeneous and rare subtype of B-cell non-Hodgkin’s lymphoma (NHL), with features of both indolent and aggressive disease. Blastoid morphology is a poor prognostic variant of MCL, and these patients rarely achieve prolonged remissions [1]. While head and neck NHLs are prevalent, the occurrence of MCL as an oropharyngeal mass is rarely reported to date. The other features of high-risk MCL include high ki67 proliferative index, p53 expression, and TP53 mutation [1]. We present a case of an oropharyngeal mass ultimately diagnosed as blastoid MCL with multiple high-risk features.

## Case presentation

**Figure 1:**
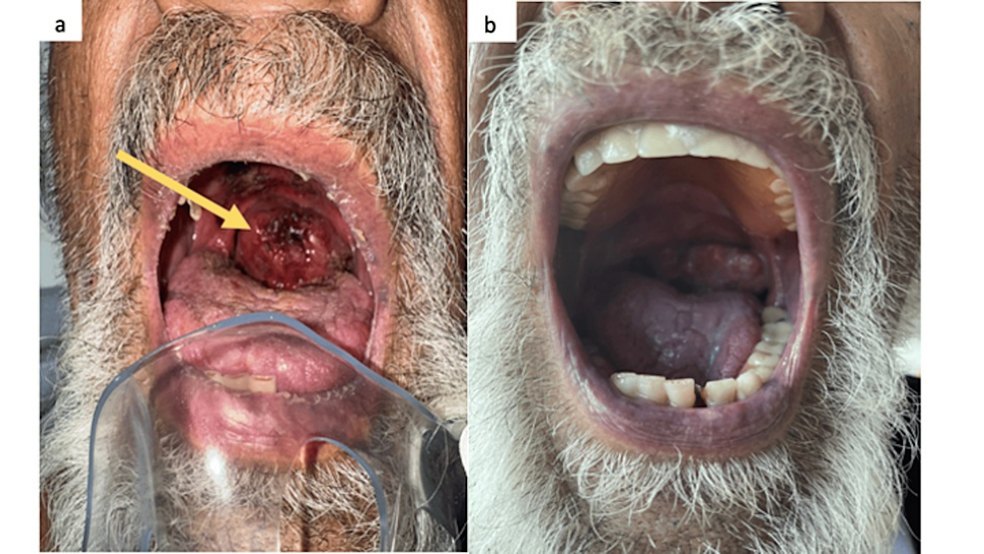
Macroscopic findings of the oropharyngeal mass (a) Oropharyngeal examination demonstrating an exophytic mass at the oropharynx with blood clots (arrow). (b) Significant mass reduction after focal radiation with a total dosage of 400 cGy in two fractions.

A 75-year-old male with a history of stroke, alcoholic cirrhosis, atrial fibrillation, and extensive smoking tobacco use of greater than 100 pack-year history, presented to the emergency room with a one-month history of dysphonia with an enlarging left-sided neck mass. He denied unintended weight loss, fever, chills, night sweats, or dysphagia. Upon further questioning, he was noted to have multiple episodes of syncope and visual hallucinations. Computed tomography (CT) scan of the brain showed no acute hemorrhage or infarct. Physical exam showed posterior oropharyngeal erythema with a large fungating mass encompassing the entire oral cavity (Figures 1a, 1b) with diffuse lymphadenopathy in anterior/cervical, supra/infraclavicular, bilateral axillary, and superficial inguinal chains including left cervical lymphadenopathy measuring 4 x 4 cm in size. No neurologic deficits were identified.

**Figure 2:**
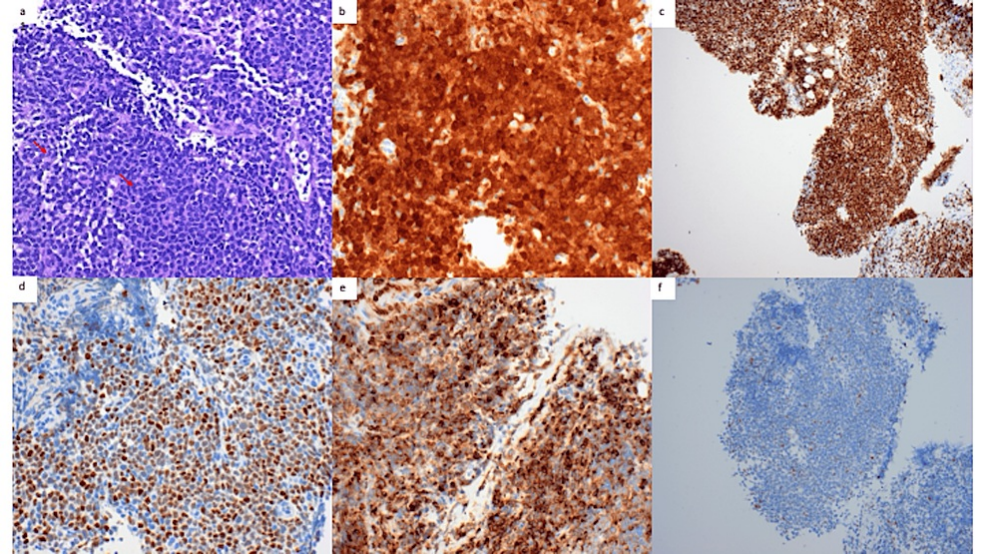
Pathological findings of the cervical lymph node (a) Hematoxylin and eosin stain at high power shows effacement of normal lymph nodal architecture by a relatively monotonous population of small to intermediate-sized lymphocytes with mitotic figures (arrows). (b) Cyclin D1 staining diffusely and strongly marking nearly all cells. (c) Ki-67 immunostaining highlighting nearly all cells. (d) SOX-11 positive staining. (e) BCL2 positive staining. (f) BCL6 negative staining. Images provided courtesy of Dr. Nigel Tourdot, Department of Pathology, University of Hawaii.

Initial work-up revealed lymphocyte-predominant leukocytosis of 12,100/μL (48% lymphocytes), normocytic normochromic anemia (hemoglobin of 11.5 g/dL), and thrombocytopenia (99,000/μL). Serum lactate dehydrogenase was elevated at 973 IU/L. Liver function tests showed an elevated AST of 43 IU/L, alkaline phosphatase 255 IU/L with normal ALT and total bilirubin. The visual hallucinations were thought to be related to hepatic encephalopathy given elevated ammonia (63 μmol/L) and chronic alcoholic cirrhosis. CT neck showed a 4.2 cm left oropharyngeal polypoid mass as well as left cervical and axillary lymphadenopathy. CT chest showed extensive left lower neck, supraclavicular, and left axillary lymphadenopathy suspicious for tumors with partially visualized lymphadenopathy in the upper abdomen. Ultrasound-guided fine needle and core biopsy of the level II lymph node on the left neck revealed MCL. Flow cytometric analysis detected a monoclonal B-cell population. Pathology showed increased mitotic and apoptotic activity and markedly increased ki-67 index (90%) and CD5-negative neoplastic lymphocytes resembling lymphoblasts, favoring an aggressive blastoid variant. The neoplastic lymphocytes were positive for CD20, PAX5, cyclin D1, SOX11, and BCL2, and negative for BCL6 ruling out small-intermediate size lymphoma (Figures 2a-2f).

CD5 is positive in 95% of cases, but this case represents the 5% of cases that are CD5 negative An adverse prognostic factor is TP53 mutation. FISH analysis was negative for deletion of 11q (ATM) with wild-type TP53, a non-mutated expression. Peripheral blood smear showed abnormal lymphocytes, consistent with the diagnosis. He was diagnosed with stage III MCL, a blastoid variant, involving the oropharynx as well as lymph nodes in the neck, axilla, upper abdomen retroperitoneum, and spleen.

Further work-up including MRI brain and lumbar puncture did not reveal any CNS involvement. EEG showed no epileptiform discharges or seizures. His syncope was believed to be related to hypoxic episodes caused by the obstructing oropharyngeal tumor. During his hospitalization, he received palliative radiation (200 cGy x 2 to the oral lesion) to the oropharyngeal lymphoma. The tumor was significantly smaller by the fourth day following the initiation of radiation and dysphonia improved. He was discharged in stable condition with minimal symptoms.

**Figure 3:**
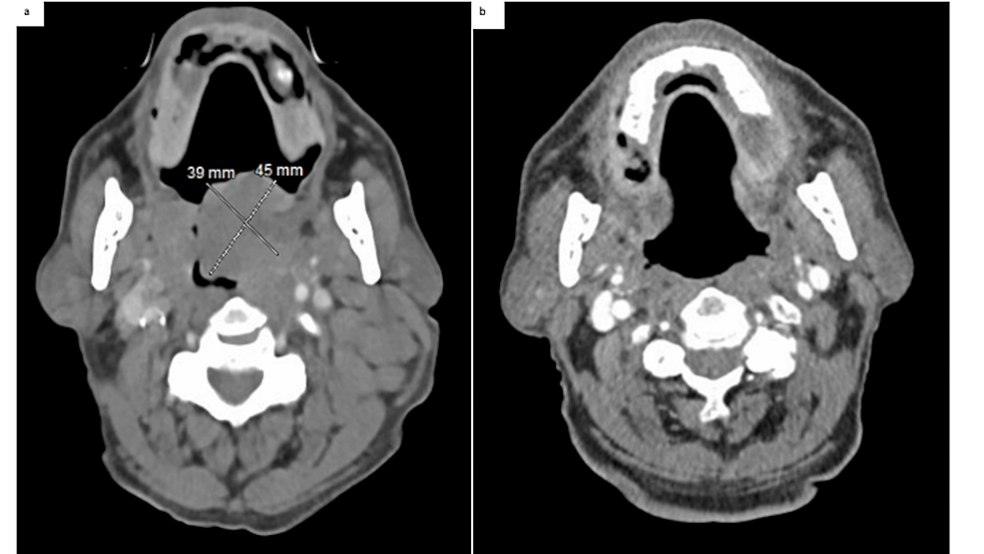
Pre- and post-treatment computed tomography (a) Enlargement of bilateral palatine tonsils, left greater than right. Left measures 4.5 x 3.9 cm. (b) Imaging post-treatment with radiation and systemic therapy (rituximab, bendamustine, and ibrutinib) showed resolution of previously reported enlargement of the palatine tonsils and bilateral cervical lymphadenopathy.

Because of the wild-type TP53 status of his lymphoma, multiple comorbidities, and the patient’s desire for a chemotherapy regimen with limited toxicity, he did not receive induction therapy with the TRIANGLE regimen consisting of alternating R-CHOP + ibrutinib/RDHAP (rituximab, cyclophosphamide, doxorubicin, vincristine, prednisone, ibrutinib)/(rituximab, dexamethasone, cytarabine) with platinum (carboplatin, cisplatin, or oxaliplatin). After two months of treatment with rituximab, bendamustine, and ibrutinib, repeat CT neck imaging showed a markedly favorable response (Figures 3a, 3b).

## Discussion

MCL represents a rare subset of B-cell non-Hodgkin lymphoma (NHL), exhibiting an annual incidence of approximately one case per 200,000 individuals. It demonstrates a predilection for men than women (3:1) with a peak incidence of 60 to 70 years old. The typical immunohistochemical staining pattern of MCL is positive for CD20, cyclin D1, CD5, and SOX11, and negative for CD10 and BCL6 [2]. Upon initial presentation, most patients tend to manifest with an advanced-stage disease, exhibiting involvement in nodal and extranodal sites such as the gastrointestinal mucosa.

The blastoid variant is an aggressive subtype with high cell proliferation (Ki-67 index > 30%). Two case reports of blastoid MCL presenting as a mass on the hard palate were documented [3]. In a retrospective study from the European MCL network, the blastoid variant was associated with a higher rate of central nervous system involvement [4]. Like the classic variant, blastoid MCL overexpresses the cyclin D1 protein with frequent mutations of ATM and TP53. While the presence of CD5 expression stands as a distinctive surface marker in MCL, a subset of patients with blastoid MCL displays an absence of CD5 expression. The subset of CD5-negative MCL was associated with indolent outcomes, which was historically misdiagnosed as marginal zone B-cell lymphoma for an extended duration, primarily due to morphological and immunophenotypic similarities, notably the absence of both CD5 and CD10 markers [2].

Treatment approaches for MCL include chemoimmunotherapy with an anthracycline-based regimen followed by high-dose therapy and autologous stem cell rescue (HDT/ASCR) for young (<65 years) and fit patients. In the TRIANGLE study, induction therapy with R-CHOP + ibrutinib/RDHAP followed by a two-year-maintenance therapy with ibrutinib and rituximab showed a higher failure-free survival rate (88% vs 72%) in young patients compared to induction therapy followed by HDT/ASCR consolidation [5]. However, longer follow-up studies are needed to ascertain the benefit of maintenance therapy over HDT/ASCR consolidation. Less aggressive therapies can be used for older patients or patients with comorbidities that would render them ineligible for anthracycline-based combinations. Radiotherapy can also provide local control and palliation in patients who cannot tolerate chemotherapy [6].

In a large, prospective, multicenter, phase 3 study, the efficacy of bendamustine plus rituximab (BR) was evaluated in 519 patients with newly diagnosed stage III or IV indolent or MCL compared to R-CHOP. BR was associated with better progression-free survival and fewer toxic effects which are significant given the aggressive course of MCL [7]. In the SHINE study of elderly patients with MCL, adding ibrutinib to BR showed a significantly prolonged PFS (80.6 vs. 52.9 months) compared to the BR plus placebo group [8]. However, due to distinct features of the blastoid variant, these patient subgroups have poorer prognoses with the standard MCL treatment. Currently, there is limited evidence to dictate the treatment of the blastoid variant due to this subtype accounting for only 5-20% of the MCL population in most clinical trials [9]. In a retrospective analysis of 58 MCL patients treated with R-CHOP, the cohort with the blastoid variant had shorter PFS (13 vs 31.7 months) compared to other MCL subtypes [10]. As in this case, determining the molecular features of MCL patients is helpful in determining prognosis, risk stratification, and choosing the appropriate treatment regimen. An enhanced understanding of the molecular pathogenesis of MCL with its unique presentation has prognostic and therapeutic implications.

## Conclusions

Blastoid MCL presenting as an oropharyngeal mass is rare. There is limited knowledge regarding its clinical course and behavior. It presents a diagnostic and therapeutic conundrum for clinicians. An accurate diagnosis entails a detailed morphologic evaluation with a comprehensive immunohistochemical panel is essential for accurately diagnosing blastoid MCL. Our case demonstrates that focal radiation therapy (total dosage of 400 cGy in two fractions) is an effective palliation for patients with oropharyngeal blastoid MCL mass presenting with hypoxia. Subsequent treatment with rituximab, bendamustine, and ibrutinib provides effective treatment.
